# Supplementary material for: Dynamic root microbiome sustains soybean productivity under unbalanced fertilization
Source: Nat Commun. 2024 Feb 23;15:1668. doi: 10.1038/s41467-024-45925-5 (PMC10891064; doi:10.1038/s41467-024-45925-5)
Supplement: Supplementary file 1 — Supplementary Information [file 41467_2024_45925_MOESM1_ESM.pdf]

Supplementary Information for

Dynamic root microbiome sustains soybean productivity under unbalanced fertilization

Mingxing Wang [a, b, #], An-Hui Ge [a, #], Xingzhu Ma [c, #], Xiaolin Wang [d], Qiujin Xie [a], Like Wang [a, b], Xianwei Song [e], Mengchen Jiang [a], Weibing Yang [a], Jeremy D. Murray [a], Yayu Wang [f], Huan Liu [f, g], Xiaofeng Cao [e, *], Ertao Wang [a, *]

[a] New Cornerstone Science Laboratory, National Key Laboratory of Plant Molecular Genetics, CAS Center for Excellence in Molecular Plant Sciences, Institute of Plant Physiology and Ecology, Chinese Academy of Sciences. Shanghai 200032, China.

[b] University of Chinese Academy of Sciences, Beijing 100049, China.

[c] Heilongjiang Academy of Black Soil Conservation and Utilization, Harbin 150086, China.

[d] College of Agriculture, South China Agricultural University, Guangzhou 510642, China.

[e] Institute of Genetics and Developmental Biology, Chinese Academy of Sciences, Beijing 100101, China.

[f] State Key Laboratory of Agricultural Genomics, Key Laboratory of Genomics, Ministry of Agriculture, BGI Research, Shenzhen 518083, China.

[g] BGI Life Science Joint Research Center, Northeast Forestry University, Harbin, 150040, China.

[#] These authors contributed equally to this article.

* To whom correspondence may be addressed. Email: etwang@cemps.ac.cn and xfcao@genetics.ac.cn.

Supplementary Figures

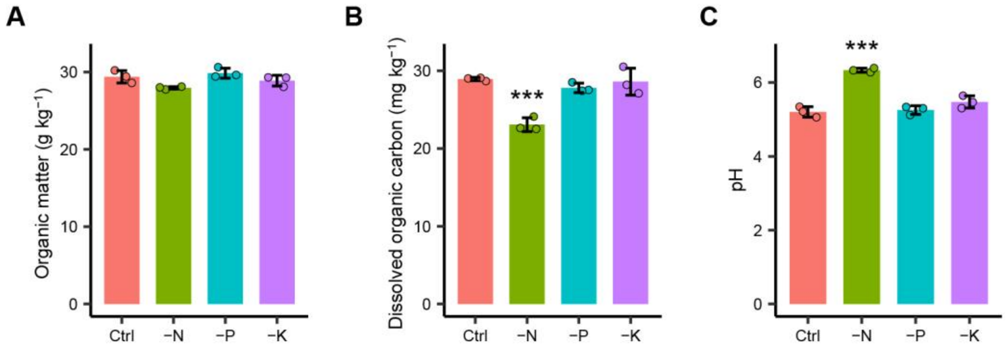

Supplementary Fig. 1 Soil chemical properties. A-C the content of soil organic matter (**A**), dissolved organic carbon (**B**) and pH value (**C**) of the bulk soils in different fertilization treatments before soybean planting in 2020 (n = 3 plots). The asterisks represent the level of significance (***P < 0.001) between Control and unbalanced fertilization treatments based on one-way ANOVA test with Dunnett's post hoc analysis (for data fit normal distributions and homogeneous variance) or Kruskal-Wallis test with Dunn's post hoc analysis (for data does not fit normal distributions or homogeneous variance). Exact P-values are listed in the Source Data file. The data are presented as mean values ± standard deviation (SD). Source data are provided as a Source Data file.

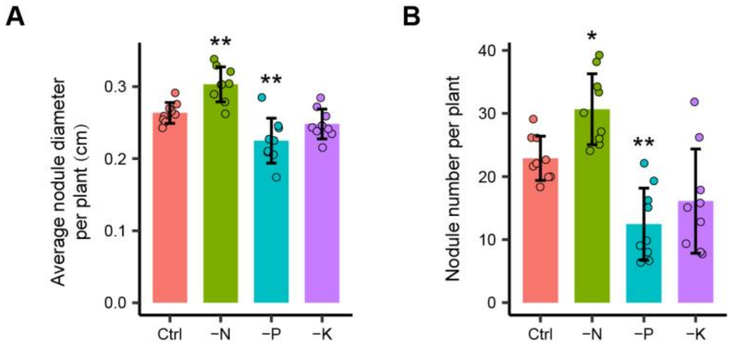

Supplementary Fig. 2 Soybean nodule phenotype. A, B effect of unbalanced fertilization on the diameter (**A**) and number (**B**) of soybean nodules counted at 42 days after seed germination (n = 9 plants). The asterisks represent the level of significance (*P < 0.05, **P < 0.01) between Control and unbalanced fertilization treatments based on one-way ANOVA test with Dunnett's post hoc analysis (for data fit normal distributions and homogeneous variance) or Kruskal-Wallis test with Dunn's post hoc analysis (for data does not fit normal distributions or homogeneous variance). Exact P-values are listed in the Source Data file. The data are presented as mean values ± standard deviation (SD). Source data are provided as a Source Data file.

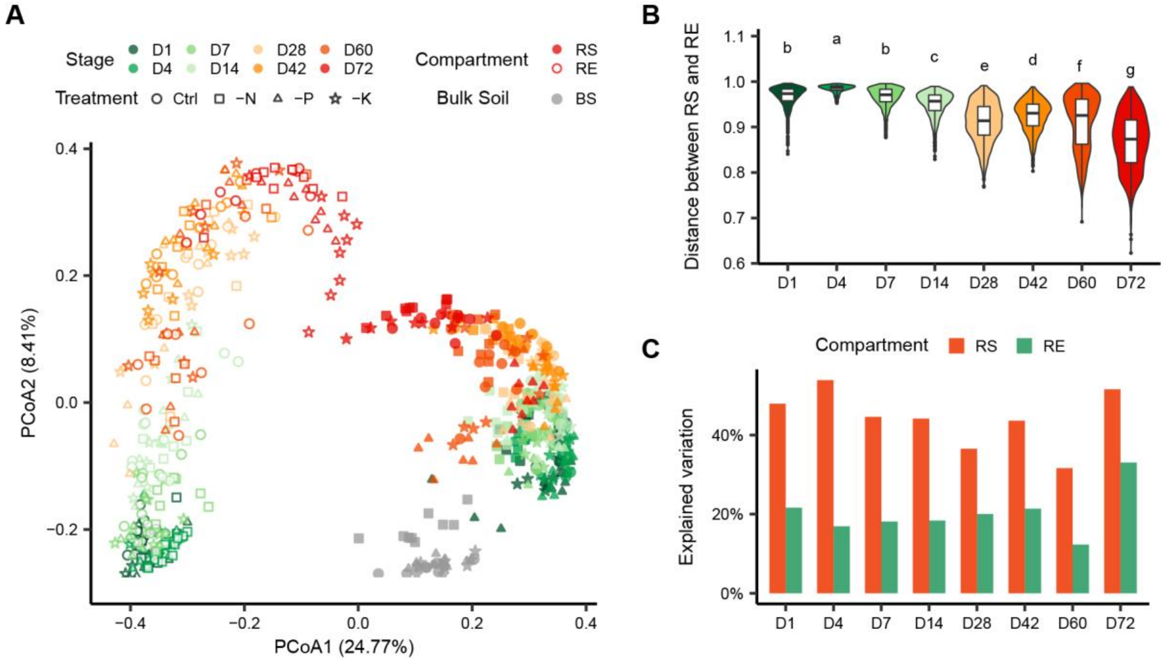

Supplementary Fig. 3 Root-associated bacterial β-diversity in different fertilization treatments by quantitative microbiome profiling (QMP). A visualized microbial β-diversity through principle coordinate analysis (PCoA) in all samples. **B** Bray-Curtis distance between the rhizosphere and root endosphere at each plant developmental stage. Different letters indicate significant difference at $P < 0.05$ by Kruskal-Wallis test with Dunn's post hoc analysis for multiple comparisons. The box plots indicate the median (center line), the 25th and 75th percentiles (box), and the range of non-outlier values (whiskers). Exact *P*-values are listed in the Source Data file. **C** explained variation of fertilization-induced change of bacterial community at each stage based on PERMANOVA in the rhizosphere (RS) and root endosphere (RE). Source data are provided as a Source Data file.

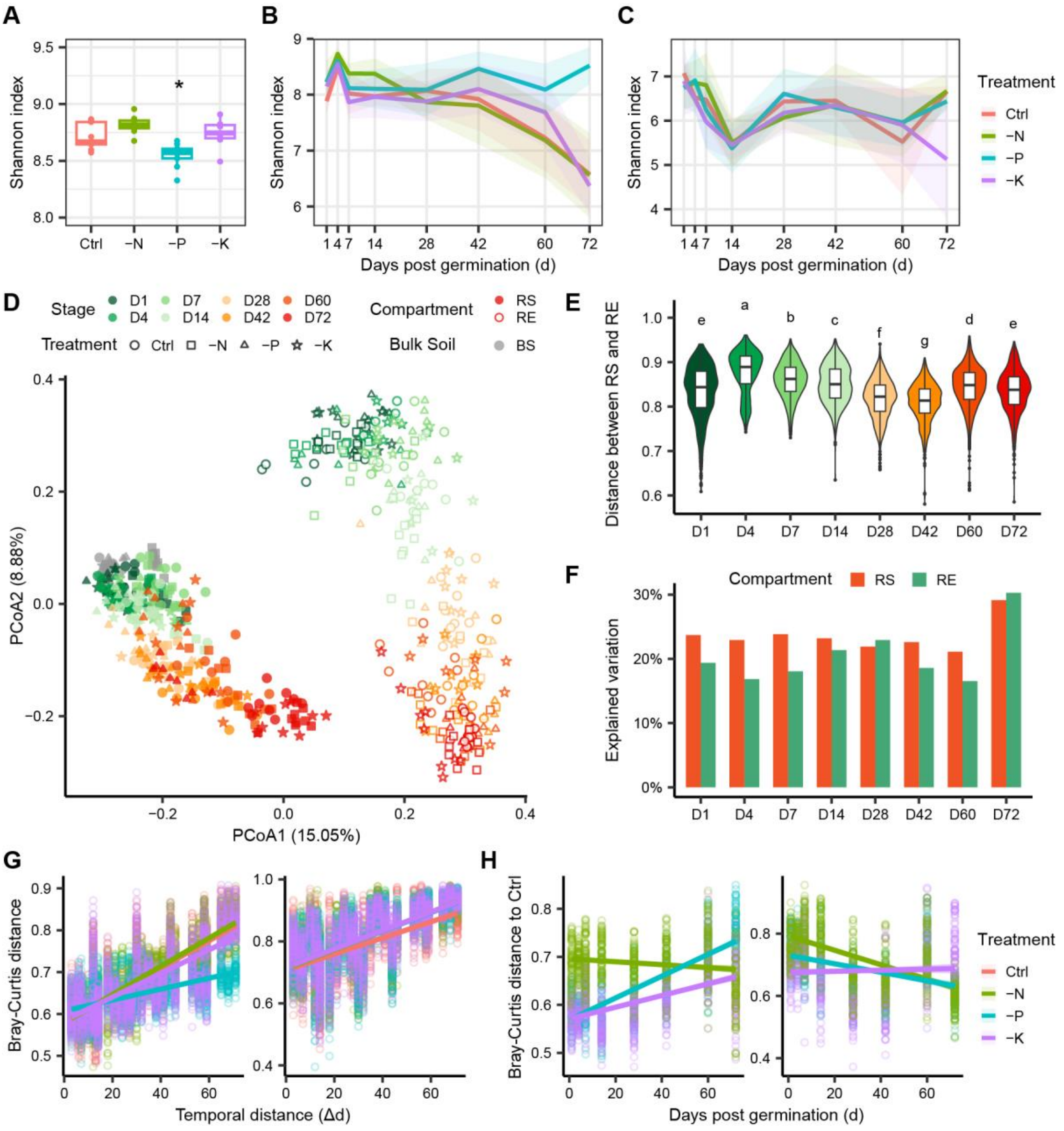

Supplementary Fig. 4 Temporal dynamics of root-associated bacterial diversity by relative microbiome profiling (RMP). A-C bacterial α-diversity (Shannon index) in the bulk soil (**A**), rhizosphere (**B**) and root endosphere (**C**). The asterisks represent the level of significance (*$P < 0.05$) between Control and unbalanced fertilization treatments based on Kruskal-Wallis test with Dunn's post hoc analysis. Exact *P*-values are listed in the Source Data file. **D** visualized microbial β-diversity through PCoA in all samples. **E** Bray-Curtis distance between rhizosphere and root endosphere. Different letters indicate significant difference at $P < 0.05$ by Kruskal-Wallis test with Dunn's post hoc analysis for multiple comparisons. Exact *P*-values are listed in the Source Data file. **F** explained variation of fertilization-induced change of bacterial community at each stage based on PERMANOVA in the rhizosphere (RS) and root endosphere (RE). **G** linear regressions between temporal distance (change of sampling day between each two samples, Δd) and Bray-Curtis distance

among samples in each treatment in the rhizosphere (left) and endosphere (right). **H** linear regressions between sampling stage (days post germination, d) and Bray-Curtis distance of each unbalanced fertilization treatment to the Control in the rhizosphere (left) and endosphere (right). The box plots (**A** and **E**) indicate the median (center line), the 25th and 75th percentiles (box), and the range of non-outlier values (whiskers). Error bands (**B** and **C**) show the standard deviation (SD). Source data are provided as a Source Data file.

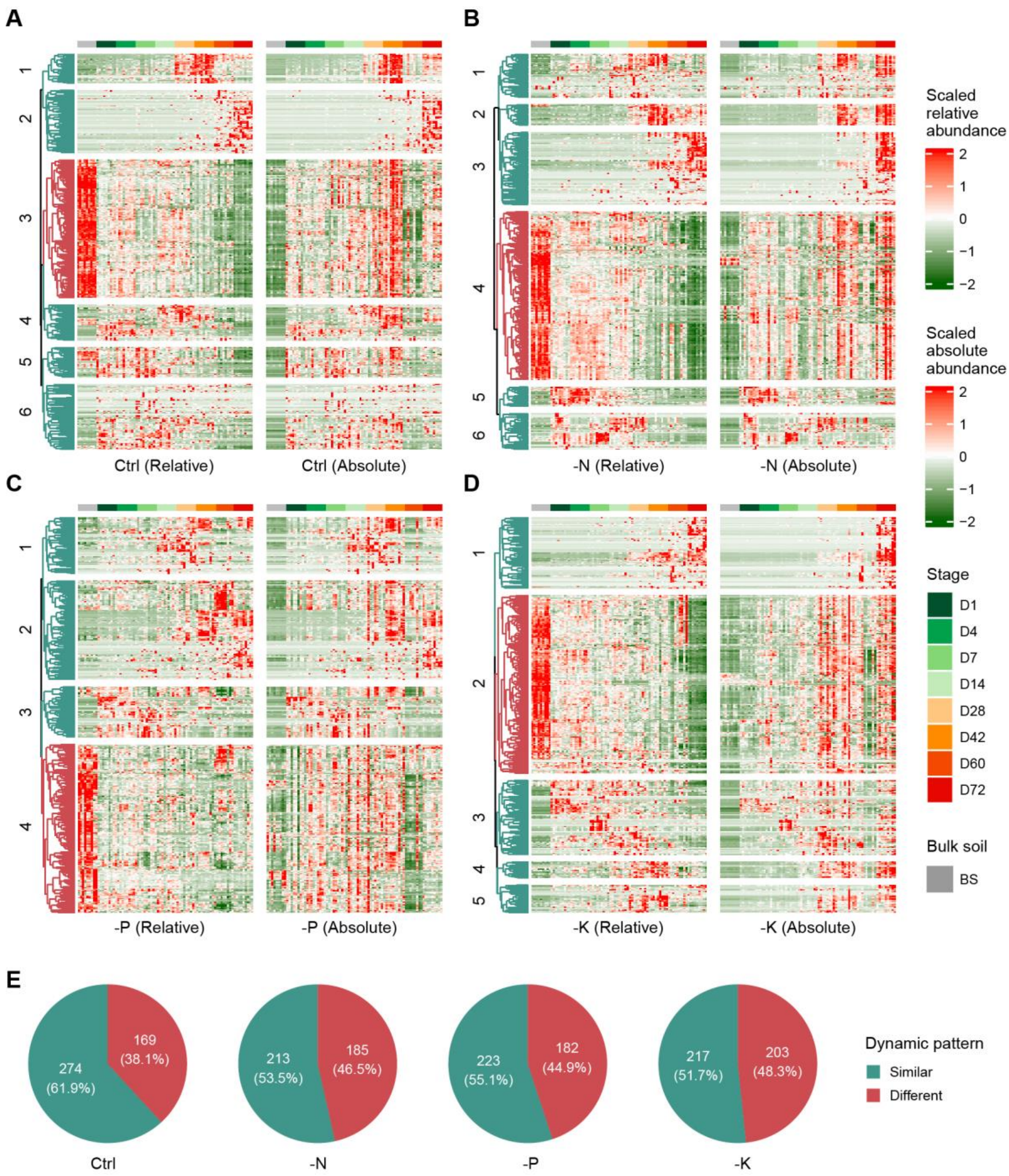

Supplementary Fig. 5 Comparison of the dynamics of rhizosphere bacteria with plant development between QMP and RMP. A-D dynamics of rhizosphere bacteria across plant developmental stages in the Control (**A**), -N (**B**), -P (**C**), and -K (**D**) treatments at ASV level (with relative abundance > 0.1% for at least one sample in each treatment). **E** statistics on the proportion of similar or different dynamic patterns of rhizosphere bacteria with plant development between QMP and RMP in different fertilization treatments. Source data are provided as a Source Data file.

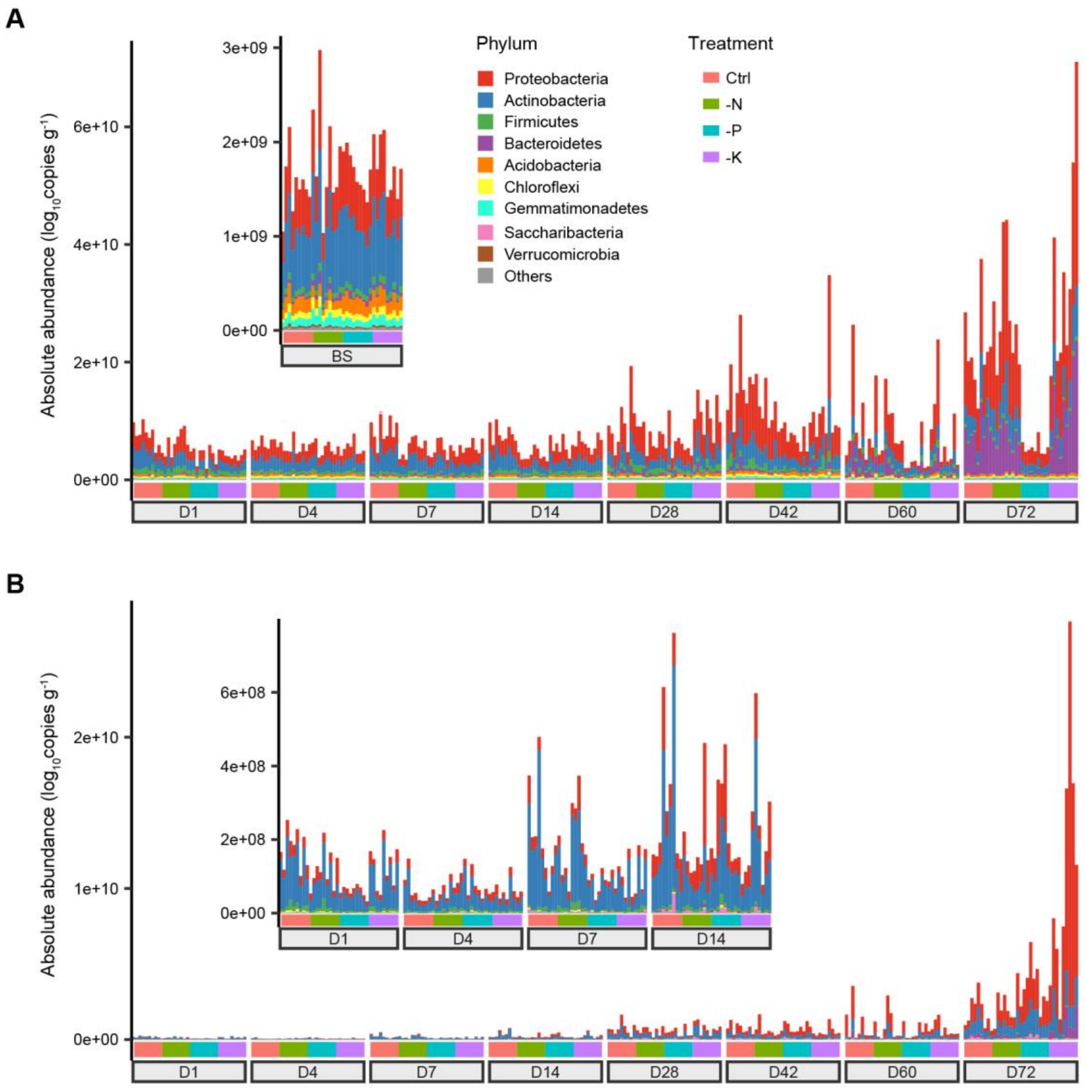

Supplementary Fig. 6 Bacterial composition at the phylum level based on QMP. A bacterial community composition in the bulk soil (BS) and rhizosphere. **B** bacterial community composition in the root endosphere. Source data are provided as a Source Data file.

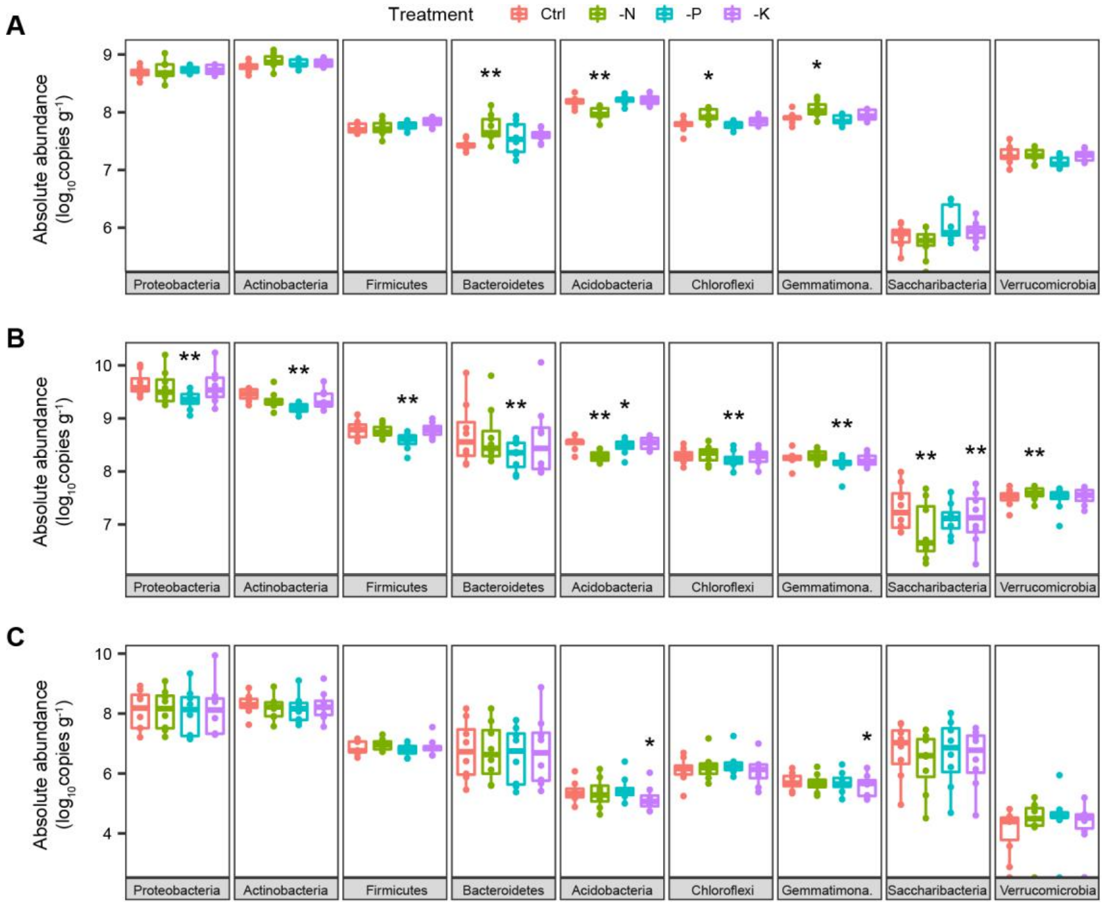

Supplementary Fig. 7 Bacterial loads of each phylum in the bulk soil, rhizosphere and root endosphere. A-C Effect of fertilization treatments on the absolute abundance of bacterial phyla in the bulk soil (**A**), rhizosphere (**B**) and root endosphere (**C**). The asterisks represent the level of significance (**P* < 0.05, ***P* < 0.01) between Control and unbalanced fertilization treatments based on paired Wilcoxon test using all samples in each compartment. Exact *P*-values are listed in the Source Data file. The box plots indicate the median (center line), the 25th and 75th percentiles (box), and the range of non-outlier values (whiskers). Source data are provided as a Source Data file.

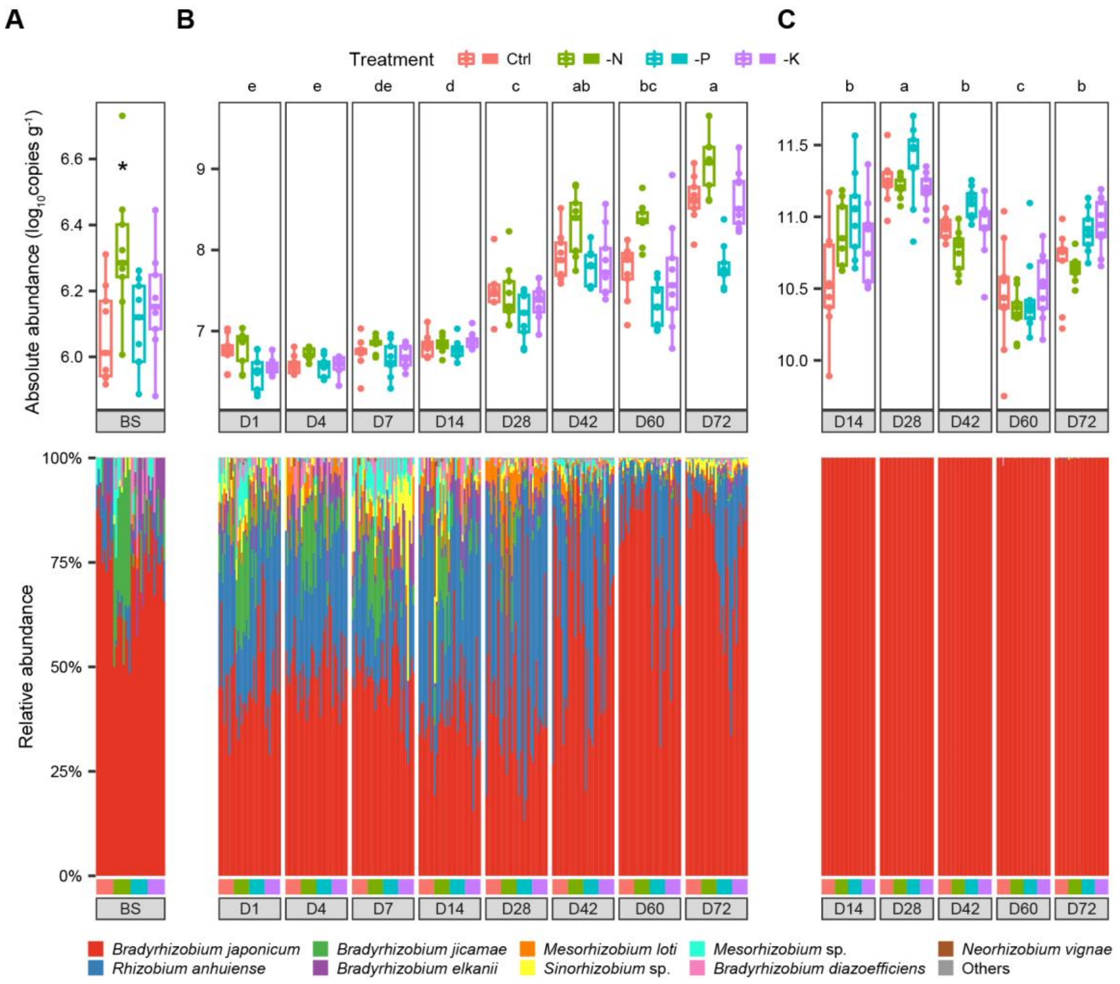

Supplementary Fig. 8 Rhizobial loads and composition in the bulk soil, rhizosphere and root endosphere based on *rpoB* sequencing data. A rhizobial abundance and community composition at species level in the bulk soil. The asterisks represent the level of significance (*$P < 0.05$) between Control and unbalanced fertilization treatments based on Kruskal-Wallis test with Dunn's post hoc analysis. **B, C** temporal dynamics of rhizobial abundance and community composition in the rhizosphere (**B**) and root endosphere (**C**). The color in the upper panel represents different treatments, and the color in the lower panel represents rhizobial species, as indicated in the accompanying legends. Different letters indicate significant difference of the average rhizobial load among developmental stages at $P < 0.05$ by Kruskal-Wallis test with Dunn's post hoc analysis for multiple comparisons. Exact *P*-values are listed in the Source Data file. The box plots indicate the median (center line), the 25th and 75th percentiles (box), and the range of non-outlier values (whiskers). Source data are provided as a Source Data file.

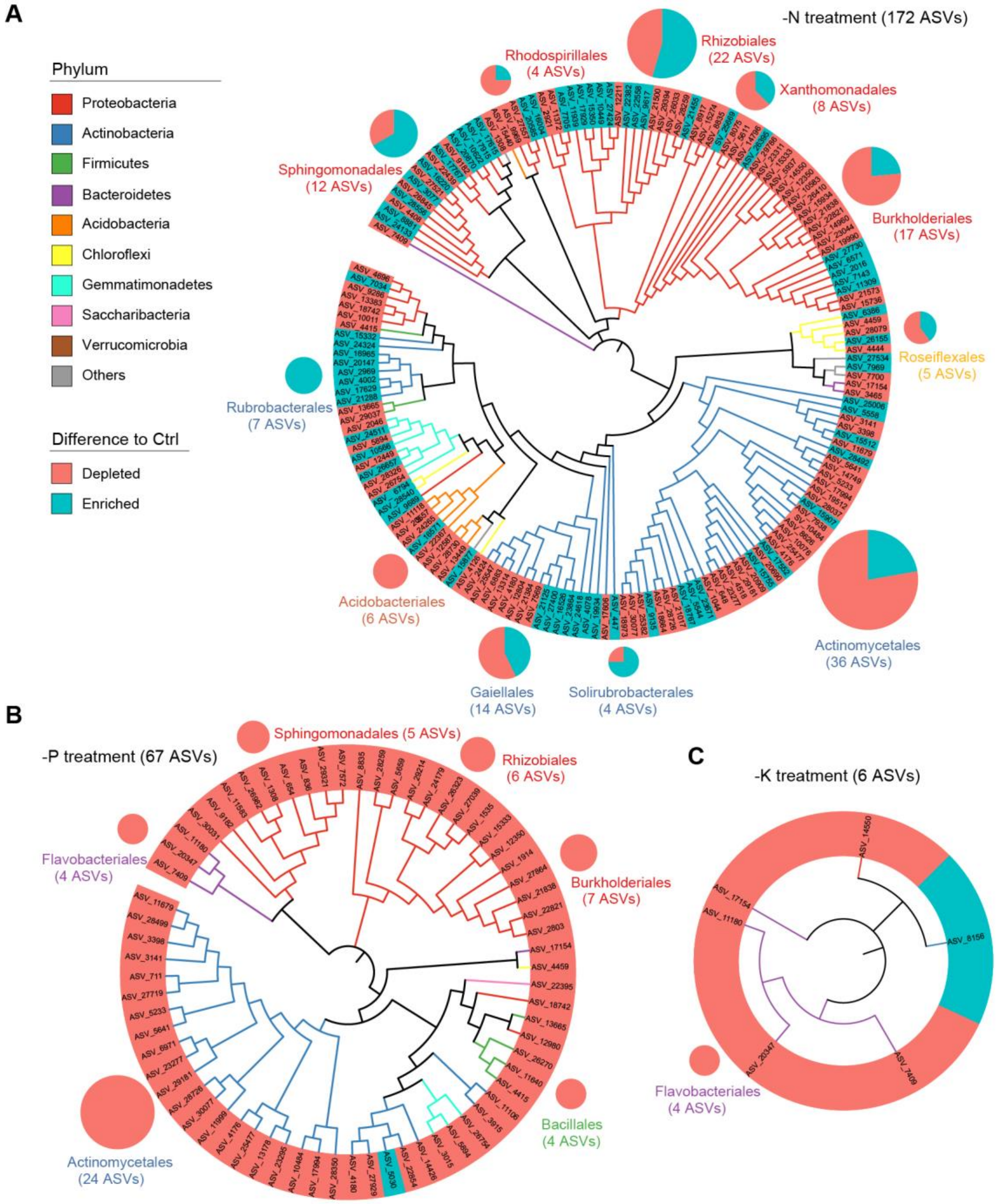

Supplementary Fig. 9 Differentiation of core ASVs between Control and each unbalanced fertilization treatment. A-C the phylogeny of ASVs with significant differences in -N (**A**), -P (**B**), and -K (**C**) treatments compared with Control. The major bacterial orders (≥ 4 ASVs) are shown in the outer ring, and the ring size is proportional to the number of ASVs. The significance of ASVs was tested by paired Wilcoxon test ($P < 0.05$). Source data are provided as a Source Data file.

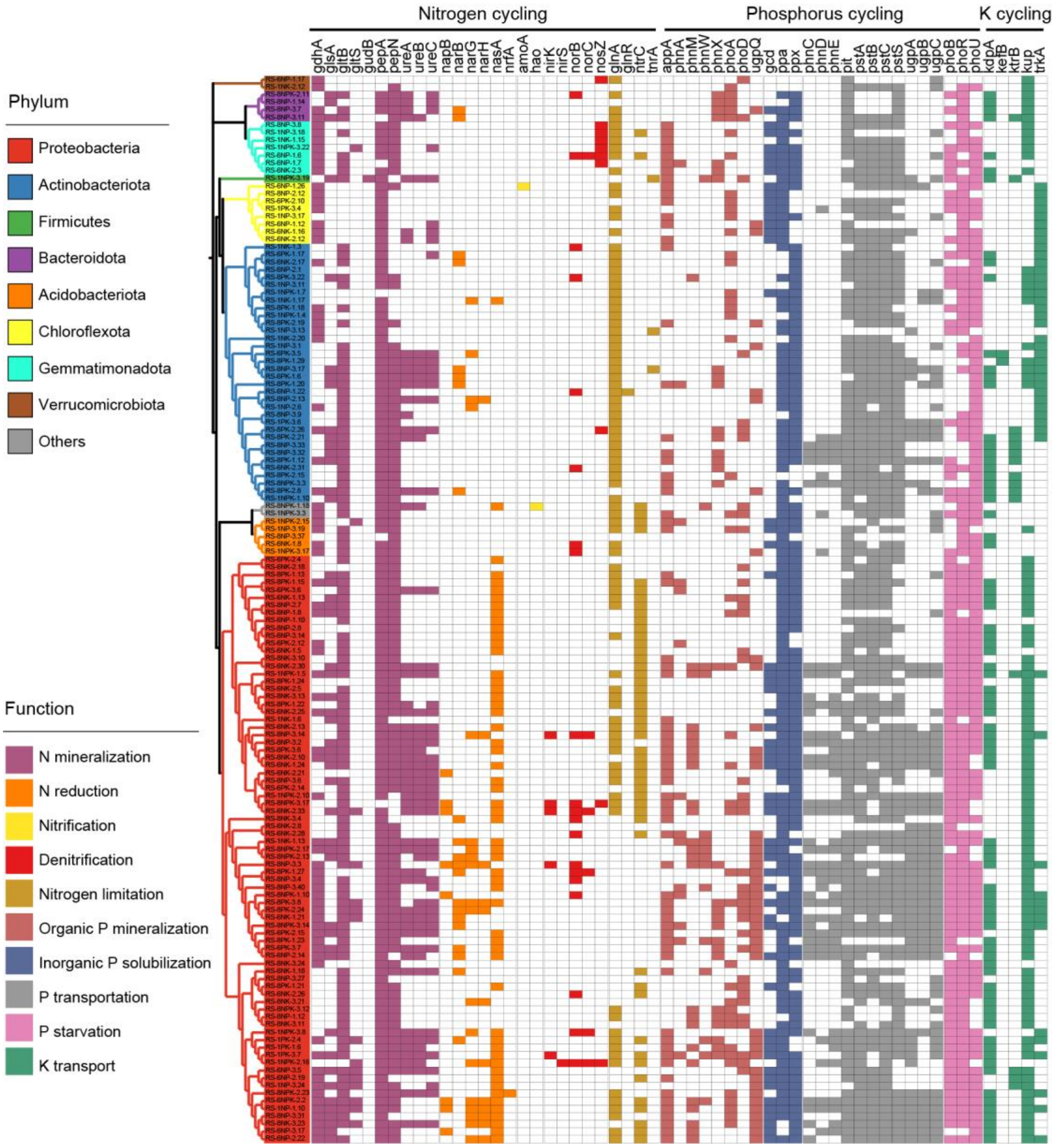

Supplementary Fig. 10 Phylogeny and functions of metagenomic assembled genomes (MAGs). The phylogenetic tree was constructed based on the complete genomes assembled from metagenomic analysis. Functional genes involved in N, P and K cycling in 140 MAGs were shown.

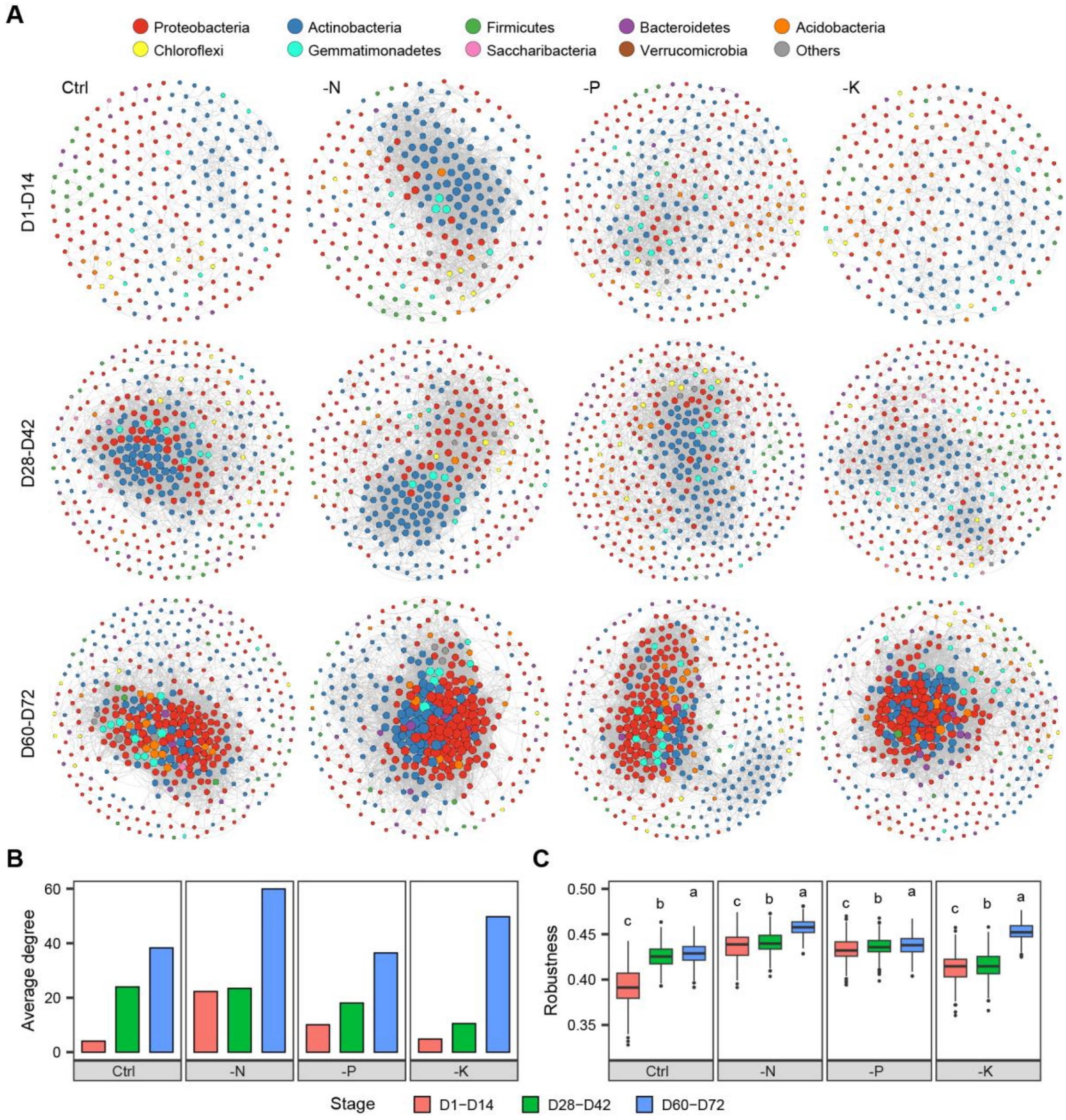

Supplementary Fig. 11 Dynamics of microbial co-occurrence networks across plant developmental stages in the rhizosphere of each treatment. **A** the co-occurrence patterns in each network. **B** dynamics of the network complexity (average degree) in different treatments. **C** dynamics of the network robustness in different treatments. Different letters indicate significant difference of the network robustness among developmental stages at $P < 0.05$ by Kruskal-Wallis test with Dunn's post hoc analysis for multiple comparisons. Exact P-values are listed in the Source Data file. The box plots indicate the median (center line), the 25th and 75th percentiles (box), and the range of non-outlier values (whiskers). Source data are provided as a Source Data file.

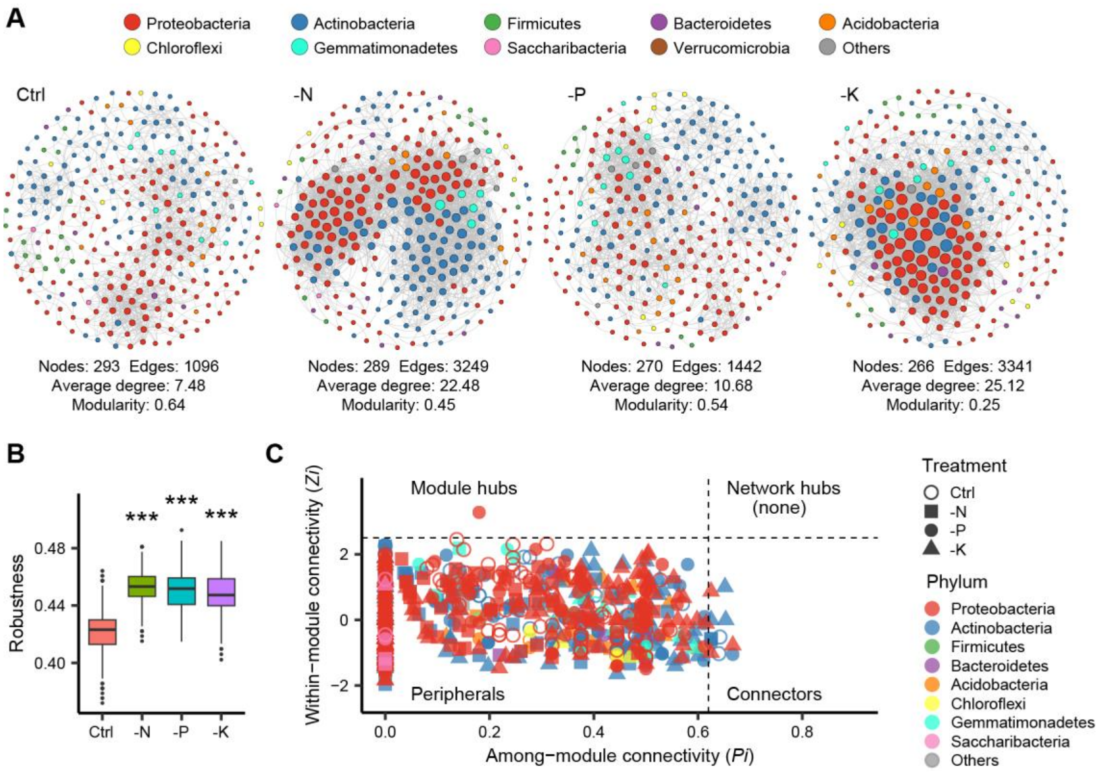

Supplementary Fig. 12 Visualized co-occurrence networks and network properties in the rhizosphere of each treatment. A the co-occurrence patterns and complexity indices in each network. **B** the robustness of each network. The asterisks represent the level of significance (***P < 0.001) between Control and unbalanced fertilization treatments based on Kruskal-Wallis test with Dunn's post hoc analysis. Exact P-values are listed in the Source Data file. The box plots indicate the median (center line), the 25th and 75th percentiles (box), and the range of non-outlier values (whiskers). **C** putative keystone hubs within the rhizosphere networks. Each symbol represents an ASV from the four networks. Nodes with Zi > 2.5 were identified as module hubs, whereas nodes with Pi > 0.62 were identified as connectors. Source data are provided as a Source Data file.

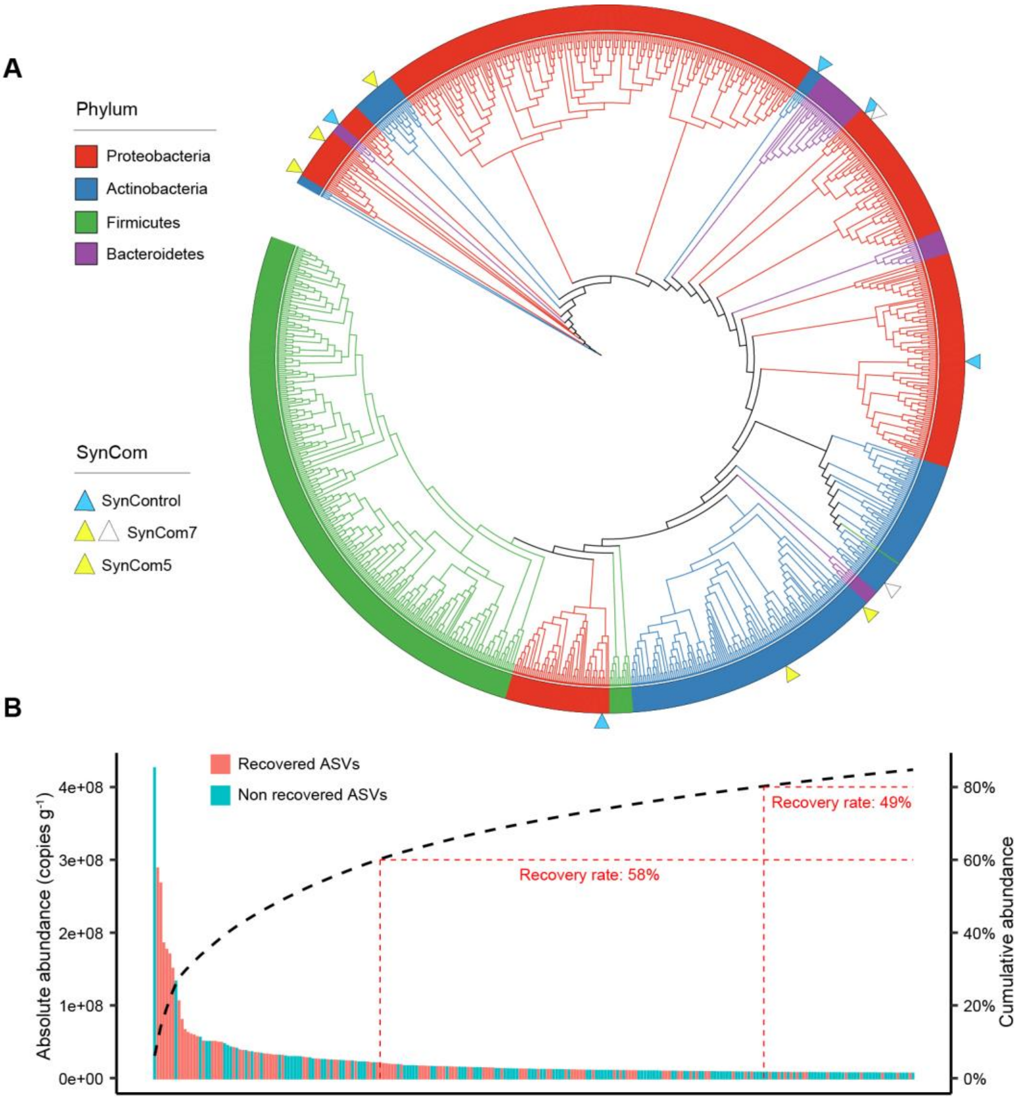

Supplementary Fig. 13 Phylogenetic tree and recovery rate of isolated bacterial strains. A evolutionary analysis of 16S rRNA gene sequences from isolated bacterial strains. **B** recovery rates of isolated bacteria at different thresholds. The rank abundance plots show the 250 most abundant core ASVs in the rhizosphere with their cumulative abundance curve. ASVs that match with a bacterial isolate (95% sequence similarity) are highlighted with red bars. The percentages of ASVs recovered as isolated strains are displayed at thresholds of 60% and 80% of accumulative absolute ASV abundances, respectively. Source data are provided as a Source Data file.

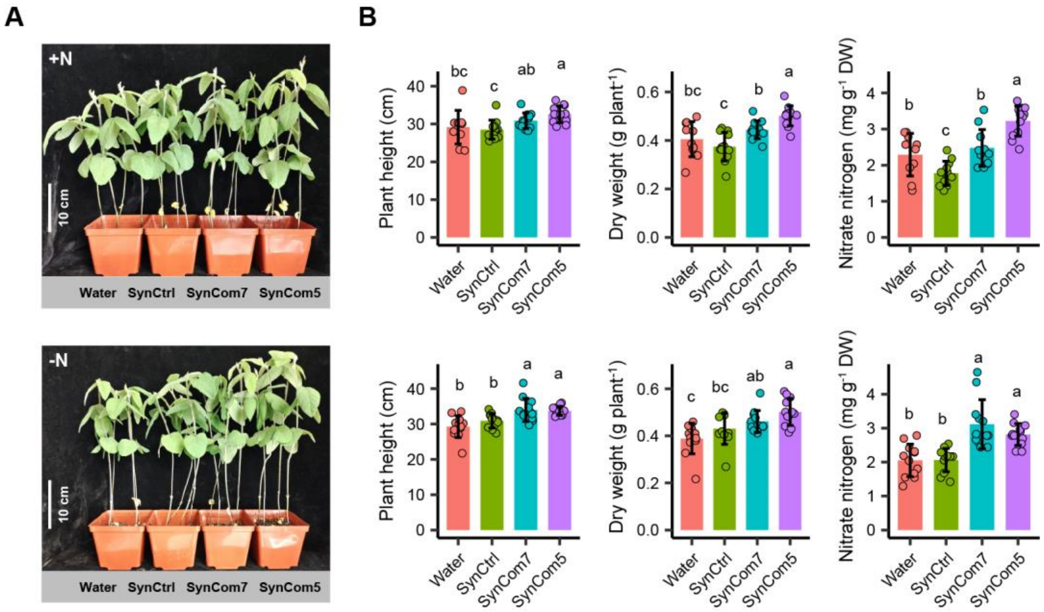

Supplementary Fig. 14 Synthetic microbial communities promote plant growth. A, B Growth phenotypes of soybeans inoculated with different SynComs after three weeks under N-amended (+N, upper panel) or N-free (-N, lower panel) conditions (n = 10 for +N Water treatment, n = 12 for other treatments). Different letters indicate significant difference of plant growth phenotypes in different treatments at $P < 0.05$ by one-way ANOVA test with LSD's post hoc analysis (for data fit normal distributions and homogeneous variance) or Kruskal-Wallis test with Dunn's post hoc analysis (for data does not fit normal distributions or homogeneous variance). Exact P-values are listed in the Source Data file. The data are presented as mean values ± standard deviation (SD). Source data are provided as a Source Data file.

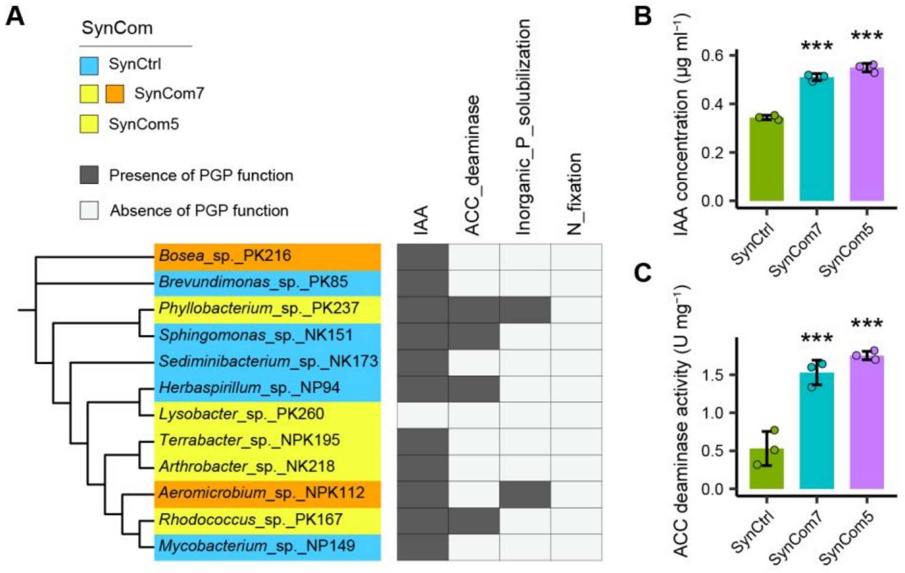

Supplementary Fig. 15 Plant growth-promoting (PGP) functions of synthetic bacterial communities. **A** the plant growth-promoting traits (i.e., IAA production, ACC deaminase activity, inorganic phosphorus solubilization, and nitrogen fixation) of each bacterial strain in the synthetic communities. **B** IAA concentration produced by each synthetic community (n = 3 biological replicates). **C** ACC deaminase activity in each synthetic community (n = 3 biological replicates). The asterisks represent the level of significance (***P < 0.001) between SynCtrl and SynCom7/SynCom5 treatments based on one-way ANOVA test with Dunnett's post hoc analysis (for data fit normal distributions and homogeneous variance) or Kruskal-Wallis test with Dunn's post hoc analysis (for data does not fit normal distributions or homogeneous variance). Exact P-values are listed in the Source Data file. The data are presented as mean values ± standard deviation (SD). Source data are provided as a Source Data file.

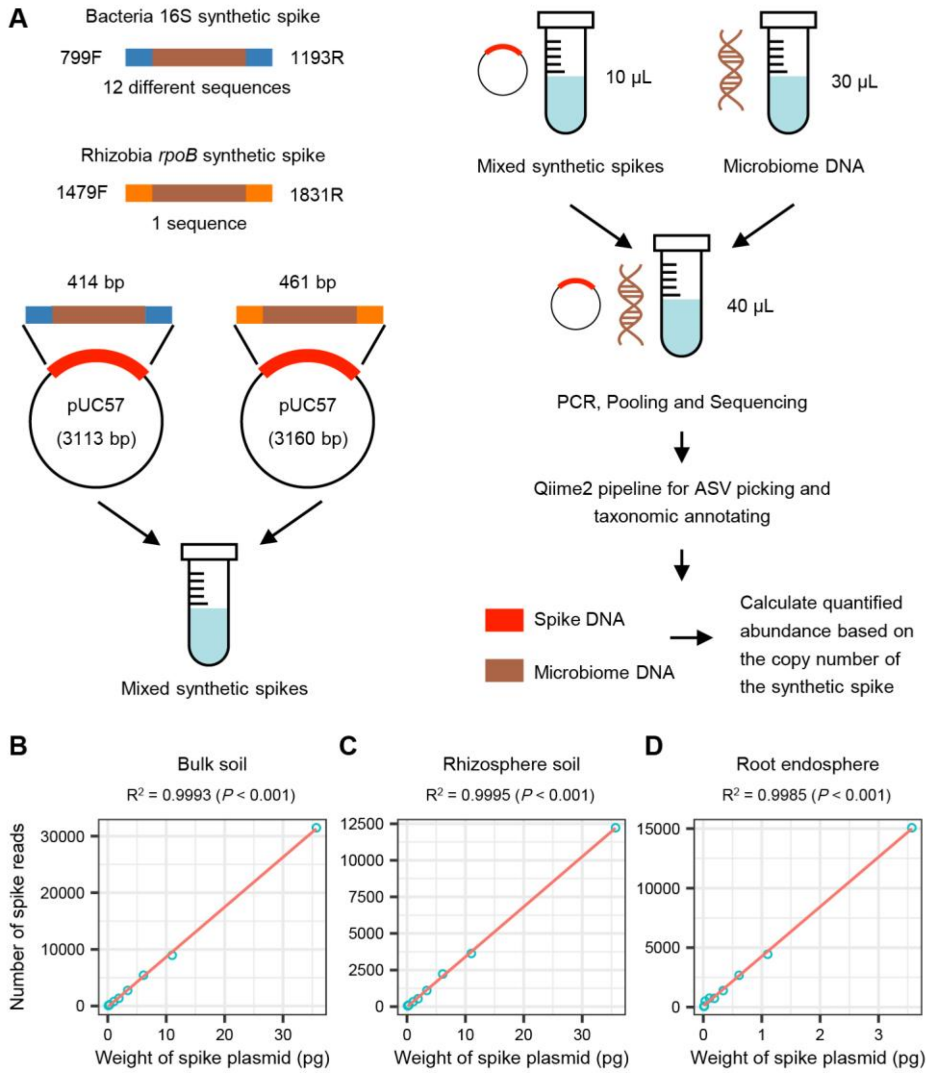

Supplementary Fig. 16 The design and test of synthetic spikes. A the procedure of synthetic spike design and experimental methods. **B-D** robustness tests of synthetic plasmids in bulk soil (**B**), rhizosphere (**C**), and root endosphere (**D**) samples.

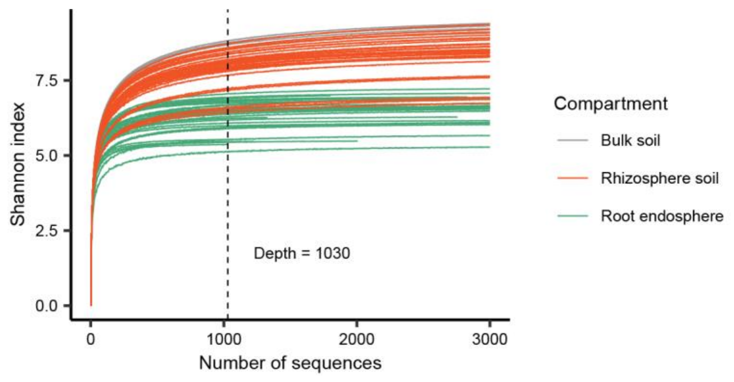

Supplementary Fig. 17 Rarefaction curves of bacterial Shannon index. Different color represents different compartments. The sampling depth is set to 1030 sequencing reads. Source data are provided as a Source Data file.

Supplementary Tables

Supplementary Table 1 The effects of root compartment, plant developmental stage, and fertilization treatment on the bacterial α-diversity based on linear mixed model (LMM). The significance level was tested by ANOVA.

	Absolute abundance		Relative abundance	
	F	*P*	*F*	*P*
Compartment	2385.3	1.9E-193	1422.0	5.8E-149
Stage	39.7	1.9E-44	36.6	2.2E-41
Treatment	13.6	1.6E-08	12.0	1.4E-07
Compartment:Stage	22.0	5.0E-26	18.1	1.6E-21
Compartment:Treatment	8.2	2.3E-05	7.0	1.3E-04
Stage:Treatment	5.7	5.4E-14	5.0	6.5E-12
Compartment:Stage:Treatment	3.0	1.3E-05	2.6	1.5E-04

Supplementary Table 2 Contribution of various factors to differences in bacterial β-diversity. The relative contribution and significant level of different factors on bacterial Bray-Curtis dissimilarity were tested with PERMANOVA.

	Absolute abundance			Relative abundance		
	R^2	*F*	*P*	R^2	*F*	*P*
Compartment	0.241	312.6	0.001	0.136	132.0	0.001
Stage	0.114	21.0	0.001	0.133	18.4	0.001
Treatment	0.040	17.4	0.001	0.045	14.5	0.001
Compartment:Stage	0.103	19.1	0.001	0.066	9.2	0.001
Compartment:Treatment	0.032	13.9	0.001	0.018	5.9	0.001
Stage:Treatment	0.043	2.6	0.001	0.049	2.3	0.001
Compartment:Stage:Treatment	0.037	2.3	0.001	0.033	1.5	0.001

Supplementary Table 3 Temporal-decay patterns of bacterial Bray-Curtis distance in different fertilization treatments. Linear regression analysis was used to examine the relationship between temporal distance and Bray-Curtis distance between two samples in each treatment, and the significance level was tested by ANOVA.

	Absolute abundance				Relative abundance			
	Rhizosphere soil		Root endosphere		Rhizosphere soil		Root endosphere	
	Slope	*P*	Slope	*P*	Slope	*P*	Slope	*P*
Ctrl	0.0043	9.0E-303	0.0024	8.0E-117	0.0032	0	0.0026	4.7E-171
-N	0.0054	0	0.0028	7.0E-184	0.0034	0	0.0030	3.4E-219
-P	0.0014	2.0E-50	0.0031	3.8E-228	0.0013	2.1E-181	0.0029	1.2E-228
-K	0.0048	2.6E-247	0.0029	3.6E-197	0.0028	0	0.0030	4.4E-259

Supplementary Table 4 Dynamics of Bray-Curtis distance between Control and unbalanced fertilization treatments across plant developmental stages. Linear regression analysis was used to examine the relationship between sampling stage and Bray-Curtis distance of each unbalanced fertilization treatment to the Control, and the significance level was tested by ANOVA.

	Absolute abundance				Relative abundance			
	Rhizosphere soil		Root endosphere		Rhizosphere soil		Root endosphere	
	Slope	*P*	Slope	*P*	Slope	*P*	Slope	*P*
-N	0.0005	4.4E-05	-0.0019	1.4E-30	-0.0003	1.2E-04	-0.0023	6.0E-65
-P	0.0040	6.9E-89	-0.0011	1.6E-11	0.0023	1.3E-133	-0.0014	2.1E-25
-K	0.0028	2.2E-62	0.0006	6.9E-04	0.0012	4.4E-47	0.0002	2.6E-01

Supplementary Table 5 The effects of root compartment, plant developmental stage, and fertilization treatment on the absolute abundance of bacteria and rhizobia based on linear mixed model (LMM). The significance level was tested by ANOVA.

	Bacteria		Rhizobia	
	F	*P*	*F*	*P*
Compartment	730.8	3.4E-100	787.0	1.1E-97
Stage	71.9	8.0E-72	36.5	1.2E-39
Treatment	21.8	2.8E-13	9.8	3.0E-06
Compartment:Stage	25.8	3.6E-30	63.5	8.4E-42
Compartment:Treatment	16.4	3.4E-10	13.0	3.9E-08
Stage:Treatment	9.7	4.3E-26	1.6	5.0E-02
Compartment:Stage:Treatment	4.7	7.3E-11	2.7	1.5E-03

Supplementary Table 6 Dynamics of the network properties in the rhizosphere of each treatment.

	D1-D14				D28-D42				D60-D72			
	Ctrl	-N	-P	-K	Ctrl	-N	-P	-K	Ctrl	-N	-P	-K
Nodes	253	253	317	258	369	332	404	369	401	343	411	407
Edges	511	2823	1597	619	4422	3888	3653	1937	7680	10283	7487	10125
Average degree	4.04	22.32	10.08	4.80	23.97	23.42	18.08	10.50	38.30	59.96	36.43	49.75
Modularity	0.77	0.29	0.49	0.70	0.21	0.37	0.36	0.49	0.26	0.17	0.28	0.15
