# Supplementary material for: Dynamic root microbiome sustains soybean productivity under unbalanced fertilization
Source: Nat Commun. 2024 Feb 23;15:1668. doi: 10.1038/s41467-024-45925-5 (PMC10891064; doi:10.1038/s41467-024-45925-5)
Supplement: Supplementary file 3 — Reporting Summary [file 41467_2024_45925_MOESM3_ESM.pdf]

nature portfolio

Corresponding author(s): NCOMMS-23-22973A
Ertao Wang, Xiaofeng Cao

Last updated by author(s): Jan 16, 2024

Reporting Summary

Nature Portfolio wishes to improve the reproducibility of the work that we publish. This form provides structure for consistency and transparency in reporting. For further information on Nature Portfolio policies, see our Editorial Policies and the Editorial Policy Checklist.

Statistics

For all statistical analyses, confirm that the following items are present in the figure legend, table legend, main text, or Methods section.

n/a	Confirmed	
☐	☒	The exact sample size (*n*) for each experimental group/condition, given as a discrete number and unit of measurement
☐	☒	A statement on whether measurements were taken from distinct samples or whether the same sample was measured repeatedly
☐	☒	The statistical test(s) used AND whether they are one- or two-sided<br>*Only common tests should be described solely by name; describe more complex techniques in the Methods section.*
☒	☐	A description of all covariates tested
☐	☒	A description of any assumptions or corrections, such as tests of normality and adjustment for multiple comparisons
☐	☒	A full description of the statistical parameters including central tendency (e.g. means) or other basic estimates (e.g. regression coefficient) AND variation (e.g. standard deviation) or associated estimates of uncertainty (e.g. confidence intervals)
☐	☒	For null hypothesis testing, the test statistic (e.g. *F*, *t*, *r*) with confidence intervals, effect sizes, degrees of freedom and *P* value noted<br>*Give P values as exact values whenever suitable.*
☒	☐	For Bayesian analysis, information on the choice of priors and Markov chain Monte Carlo settings
☒	☐	For hierarchical and complex designs, identification of the appropriate level for tests and full reporting of outcomes
☐	☒	Estimates of effect sizes (e.g. Cohen's *d*, Pearson's *r*), indicating how they were calculated

Our web collection on statistics for biologists contains articles on many of the points above.

Software and code

Policy information about availability of computer code

Data collection	The V5-V7 region of bacterial 16S rRNA gene, and rpoB gene of symbiotic rhizobia were amplified through primer pairs 799F (5'-AAC MGG ATT AGA TAC CCK G-3') and 1193R (5'-ACG TCA TCC CCA CCT TCC-3') , and rpoB1479F (5'-GAT CGA RAC GCC GGA AGG-3') and rpoB1831R (5'-TGC ATG TTC GAR CCC AT-3') , respectively. The resultant 16S rRNA and rpoB derived PCR products were sequenced on the MGISEQ-2000 platform of BGISEQ using the single-end 400 bp (SE400) module in BGI Shenzhen (BGI, Shenzhen, China). The rhizosphere and endosphere samples from days 1, 42 and 72 after germination were subjected to metagenomic sequencing on the DNBSEQ-T7 platform of BGISEQ using the paired-end 100 bp (PE100) module in BGI Shenzhen (BGI, Shenzhen, China).
Data analysis	The microbial bioinformatic analysis was performed with QIIME 2 2021.11. The raw sequencing data was demultiplexed and filtered using the q2-demux plugin followed by denoising with DADA2. The metagenomic raw reads were filtered to remove low-quality and host (Glycine max) genomic sequences using KneadData (v0.10.0). Metagenome assembly of individual samples was processed by MEGAHIT (v1.2.9) using the default parameters. Bowtie 2 (v2.4.4) was used to map the raw reads to assembled contigs to calculate the read coverage of each contig, after evaluating the quality of contigs by QUAST (v5.0.2). The contigs were subjected open reading frame (ORF) prediction using Prodigal (v2.6.3). Afterwards, a non-redundant gene catalogue was constructed using CD-HIT (v4.8.1). The raw reads and normalized TPM (Transcripts Per kilobase Million) data was quantified after BLAST against the non-redundant gene catalogue by Salmon (v1.6.0). The non-redundant genes were annotated using GO, KEGG, and COG databases by eggNOG-mapper (v2.1.6), and CAZy database using DIAMOND.

For manuscripts utilizing custom algorithms or software that are central to the research but not yet described in published literature, software must be made available to editors and reviewers. We strongly encourage code deposition in a community repository (e.g. GitHub). See the Nature Portfolio guidelines for submitting code & software for further information.

Data

Policy information about availability of data

All manuscripts must include a data availability statement. This statement should provide the following information, where applicable:

- Accession codes, unique identifiers, or web links for publicly available datasets
- A description of any restrictions on data availability
- For clinical datasets or third party data, please ensure that the statement adheres to our policy

The 16S rRNA amplicon data (SAMN32647504-SAMN32648110), rpoB amplicon data (SAMN32650334-SAMN32650836), and metagenome data (SAMN32727371-SAMN32727406) associated with this study have been deposited in the NCBI Sequence Read Archive (SRA) under the project accession PRJNA922226 (https://www.ncbi.nlm.nih.gov/bioproject/PRJNA922226). The classification information of the metagenomic assembled genomes (MAGs) and isolated bacterial strains has been uploaded to the GitHub repository: https://github.com/mxwang2016/Soybean. Source data are provided with this paper.

Research involving human participants, their data, or biological material

Policy information about studies with human participants or human data. See also policy information about sex, gender (identity/presentation), and sexual orientation and race, ethnicity and racism.

Reporting on sex and gender	n/a
Reporting on race, ethnicity, or other socially relevant groupings	n/a
Population characteristics	n/a
Recruitment	n/a
Ethics oversight	n/a

Note that full information on the approval of the study protocol must also be provided in the manuscript.

Field-specific reporting

Please select the one below that is the best fit for your research. If you are not sure, read the appropriate sections before making your selection.

☐ Life sciences ☐ Behavioural & social sciences ☒ Ecological, evolutionary & environmental sciences

For a reference copy of the document with all sections, see nature.com/documents/nr-reporting-summary-flat.pdf

Ecological, evolutionary & environmental sciences study design

All studies must disclose on these points even when the disclosure is negative.

Study description	The long-term field experiment launched in 1979 was located at Daowai District, Harbin, China (126°51′ E, 45°50′ N). The climate is typical mid-temperate continental monsoon, and the soil type is classified as Mollisol. Given that the crop can only be cultivated once a year, a three-year soybean-maize-wheat rotation system was adopted. Four treatments were set, including (1) full-dose fertilization (NPK fertilizer, Control); (2) lack of nitrogen fertilizer (-N); (3) lack of phosphorous fertilizer (-P); and (4) lack of potassium fertilizer (-K). Each treatment consisted of three plots with a completely randomized block design, and each plot comprised 36 m2. The nitrogen fertilizer used in the experiment was urea, the phosphorous fertilizer was a combination of Ca(H2PO4)2·CaHPO4 and (NH4)2HPO4, and the potassium fertilizer used was K2SO4. For soybean cropping, the application rates were 75 kg ha-1, 150 kg ha-1 and 75 kg ha-for N, P2O5 and K2O, respectively. The field management included irrigation and weeding and was identical for the different treatments. The rhizosphere soil and root samples were collected from the soybean plots in the 2020 planting season. Before sowing, the bulk soils of each treatment were sampled as the background. The soybean variety "Heinong 84" was sown in early May and it germinated after ten days. We collected the rhizosphere soil and corresponding root samples at 1, 4, 7, 14, 28, 42, 60 and 72 days after germination.
Research sample	Overall, there are 36 bulk soil samples, 287 rhizosphere soil samples, 284 endosphere root samples, 180 nodule samples, and 36 rhizosphere soil samples for metagenomic sequencing.
Sampling strategy	The rhizosphere soil and root samples were collected from the soybean plots in the 2020 planting season. Before sowing, the bulk soils of each treatment were sampled as the background. We collected the rhizosphere soil and corresponding root samples at 1, 4, 7, 14, 28, 42, 60 and 72 days after germination. For each plot, three plants with similar growth state were randomly chosen for collection, and the sampling location was apart from previous sampling stages to prevent the potential marginal effect and disturbance with adjacent plants, with 9 biological samples for each treatment at each stage.
Data collection	The resultant 16S rRNA and rpoB derived PCR products were sequenced on the MGISEQ-2000 platform of BGISEQ using the single-

Data collection	end 400 bp (SE400) module in BGI Shenzhen (BGI, Shenzhen, China). The rhizosphere and endosphere samples from days 1, 42 and 72 after germination were subjected to metagenomic sequencing on the DNBSEQ-T7 platform of BGISEQ using the paired-end 100 bp (PE100) module in BGI Shenzhen (BGI, Shenzhen, China).
Timing and spatial scale	The long-term field experiment launched in 1979 was located at Daowai District, Harbin, China (126°51′ E, 45°50′ N). The soybean sampling season ranged from May to August in 2020.
Data exclusions	no data were excluded from the analyses.
Reproducibility	There are 9 biological repeats in amplicon samples, 3 biological repeats for metagenomic sequencing, and 12 biological repeats in synthetic community experiment.
Randomization	Each treatment consisted of three plots with a completely randomized block design in the field study.
Blinding	Blinding was not applicable.

Did the study involve field work? ☒ Yes ☐ No

Field work, collection and transport

Field conditions	The long-term field experiment launched in 1979 was located at Daowai District, Harbin, China (126°51′ E, 45°50′ N). The climate is typical mid-temperate continental monsoon, and the soil type is classified as Mollisol. Given that the crop can only be cultivated once a year, a three-year soybean-maize-wheat rotation system was adopted. Four treatments were set, including (1) full-dose fertilization (NPK fertilizer, Control); (2) lack of nitrogen fertilizer (-N); (3) lack of phosphorous fertilizer (-P); and (4) lack of potassium fertilizer (-K). Each treatment consisted of three plots with a completely randomized block design, and each plot comprised 36 m2. Different plots were separated by concrete walls (0.15 m in width and 1.1 m in depth).
Location	The long-term field experiment was located at Daowai District, Harbin, China (126°51′ E, 45°50′ N).
Access & import/export	The field trial station belongs to Heilongjiang Academy of Agricultural Science, China. We are granted access to this location for field experiments under the accompany with a staff member from the institution, who is also a co-author in our study.
Disturbance	The field management included irrigation and weeding and was identical for the different treatments. And the field was not disturbed by other environments.

Reporting for specific materials, systems and methods

We require information from authors about some types of materials, experimental systems and methods used in many studies. Here, indicate whether each material, system or method listed is relevant to your study. If you are not sure if a list item applies to your research, read the appropriate section before selecting a response.

Materials & experimental systems

n/a	Involved in the study	
☒	☐	Antibodies
☒	☐	Eukaryotic cell lines
☒	☐	Palaeontology and archaeology
☒	☐	Animals and other organisms
☒	☐	Clinical data
☒	☐	Dual use research of concern
☐	☒	Plants

Methods

n/a	Involved in the study	
☒	☐	ChIP-seq
☒	☐	Flow cytometry
☒	☐	MRI-based neuroimaging

Dual use research of concern

Policy information about dual use research of concern

Hazards

Could the accidental, deliberate or reckless misuse of agents or technologies generated in the work, or the application of information presented in the manuscript, pose a threat to:

No	Yes	
☒	☐	Public health
☒	☐	National security
☒	☐	Crops and/or livestock
☒	☐	Ecosystems
☒	☐	Any other significant area

Experiments of concern

Does the work involve any of these experiments of concern:

No	Yes	
☒	☐	Demonstrate how to render a vaccine ineffective
☒	☐	Confer resistance to therapeutically useful antibiotics or antiviral agents
☒	☐	Enhance the virulence of a pathogen or render a nonpathogen virulent
☒	☐	Increase transmissibility of a pathogen
☒	☐	Alter the host range of a pathogen
☒	☐	Enable evasion of diagnostic/detection modalities
☒	☐	Enable the weaponization of a biological agent or toxin
☒	☐	Any other potentially harmful combination of experiments and agents

Plants

Seed stocks	All soybean seeds were collected from the field in Daowai District, Harbin, China (126°51′ E, 45°50′ N) in 2019.
Novel plant genotypes	n/a
Authentication	The seeds are owned by the Heilongjiang Academy of Agricultural Sciences, and we have their permission to use them.
